# Editorial: Cardiovascular genetics—focus on paediatric cardiomyopathy

**DOI:** 10.3389/fped.2023.1147527

**Published:** 2023-02-10

**Authors:** Emanuele Monda, Juan Pablo Kaski, Giuseppe Limongelli

**Affiliations:** ^1^ Inherited and Rare Cardiovascular Diseases, Department of Translational Medical Sciences, University of Campania “Luigi Vanvitelli”, Monaldi Hospital, Naples, Italy; ^2^ Centre for Paediatric Inherited and Rare Cardiovascular Disease, Institute of Cardiovascular Science, University College London, London, United Kingdom

**Keywords:** cardiomyopathy, hypertrophic cardiomyopathy, dilated cardiomyopathy, arrthythmogenic right ventricular cardiomyopathy, restrictive cardiomyopathy

**Editorial on the Research Topic** Cardiovascular genetics—focus on paediatric cardiomyopathy

Cardiomyopathies (CMPs) are a large group of disorders characterized by structural, functional, and electrical abnormalities of the heart muscle (1). These conditions are rare in childhood but associated with increased mortality and morbidity (1). Over the last few years, data on the genetic basis, natural history, risk stratification, and treatment of childhood CMPs have emerged.

In this Research Topic, experts in the field explored this poorly characterised area with comprehensive and state of the art reviews, original articles, and challenging case reports, dealing with molecular basis, role of genetic testing, clinical manifestations, outcome, and management of childhood CMPs.

This Research Topic collected 14 articles, and we are pleased to introduce them to readers of *Frontiers in Pediatrics*.

## Cardiomyopathies

Childhood CMPs include a large spectrum of diseases, with outcome mainly depending on phenotype, aetiology, and clinical characteristics (2). CMPs are generally categorized into 5 main phenotypes, namely hypertrophic (HCM), dilated (DCM), restrictive (RCM), arrhythmogenic (ACM), and left ventricular non-compaction (LVNC) cardiomyopathy (3). In this regard, Rath et al. provided an overview of childhood CMPs, with a focus on epidemiology, natural history, and outcomes according to clinical phenotype and aetiology.

Recently, it has been observed that morphological, functional, and electrical abnormalities of the heart muscle may be part of the spectrum of Marfan syndrome and heritable thoracic aortic disease (4), contributing to morbidity and mortality of these patients. In their review, Laura Muiño-Mosquera et al. reported data on preclinical studies (i.e., mouse models of Marfan syndrome) and described the clinical presentation of cardiovascular involvement of children and adults with genetic aortic diseases.

## Hypertrophic cardiomyopathy

HCM is a myocardial disease characterized by increased left ventricular wall thickness not solely explained by abnormal loading conditions (5). It represents the second most common cause of CMP presenting in childhood. The aetiology of childhood HCM is more heterogenous than seen in the adult population (6), and includes genetic syndromes (e.g., RASopathies) (7), inborn errors of metabolism (8), and neuromuscular disorders (9).

Among the different causes of HCM in childhood, mutations in sarcomeric genes account for the large majority of cases. Norrish et al. provided a comprehensive review of sarcomeric HCM in children, highlighting the variability in disease expression and unanswered questions. In contrast, Monda et al. presented a detailed review of the non-sarcomeric causes of HCM in children, focusing on their pathophysiology, clinical features, diagnosis, and etiological therapy.

In the large spectrum of HCM aetiologies, inborn errors of metabolism represent an extremely rare cause. For example, methylmalonic acidaemia is an inherited disease caused by mutations in different genes, which result in impairment of methylmalonyl-CoA mutase or impaired intracellular cobalamin transport and processing (10). Large studies describing the cardiac involvement of patients with methylmalonic acidaemia are lacking. Thus, Liu et al. screened 99 patients with methylmalonic acidaemia using electrocardiography and echocardiography. In their original article, the authors described the prevalence of congenital heart disease, cardiomyopathy, and pulmonary hypertension, and detailed electrocardiographic ed echocardiographic characteristics of patients with this rare life-threatening disease.

Exome sequencing demonstrated a high diagnostic yield in achieving a genetic diagnosis of rare disorders (11), and its role in the diagnosis of rare CMPs is expanding (12). In this Research Topic, two well-characterized case reports of patients with rare mitochondrial disorders associated with HCM diagnosed using exome sequencing are described. Wang et al. described two cases of Sengers syndrome, a rare autosomal recessive disorder due to mutations in acylglycerol kinase (AGK) gene. Both cases exhibited the classic clinical presentation of Sengers syndrome, including HCM, bilateral cataract, myopathy, and lactic acidosis. Furthermore, they provided a comprehensive literature systematic review of previously published cases and discussed the importance of exome sequencing for the diagnosis of rare genetic diseases. Moreover, Wang et al. described the cases of a 14-year-old boy with HCM, exercise intolerance, ptosis, and lactic acidosis, and his 9-year-old brother with similar clinical feature. Using exome sequencing, these two patients were diagnosed with early onset combined oxidative phosphorylation deficiency 33 (COXPD33), a rare mitochondrial disease caused by a homozygous mutation in the C1QBP gene. Finally, Wang et al. in their mini-review, summarized the physiological function of complement C1q binding protein (C1QBP) and its mutation-associated mitochondrial CMP.

## Dilated cardiomyopathy

Dilated cardiomyopathy is a myocardial disease characterized by left ventricular dilation and dysfunction not solely explained by congenital heart disease, valve disease, or coronary artery disease (13). DCM is the most common cause of childhood CMP and is associated with a high risk for mortality (14). Several varieties of causation have been described for DCM and are generally classified as genetic and non-genetic causes (1). Among genetic causes of childhood DCM, a rare cause is represented by neuromuscular disorders (NMDs). NMDs may be inherited in autosomal dominant or recessive, or X-linked recessive patterns. Emery-Dreifuss muscular dystrophy is dominated by skeletal muscle weakness and cardiac involvement. However, cardiac abnormalities rarely appear in paediatric age. Kovalchuk et al. described five patients with different forms of Emery-Dreifuss muscular dystrophy caused by mutations in EMD or LMNA genes, presented with early-onset of cardiac abnormalities and no prominent skeletal muscle phenotype.

The pathophysiology of DCM is complex and not fully elucidated. Recently, it has been reported that long non-coding RNAs (lncRNAs) play a crucial role in regulating disease presentation and severity of several cardiovascular diseases (15). Cai et al. evaluated the expression profile of lncRNAs in children with DCM and explored their possible function, providing new relevant perspective for future research.

## Arrhythmogenic cardiomyopathy

Arrhythmogenic right ventricular cardiomyopathy (ARVC) is a myocardial disease characterized by ventricular arrhythmias and ventricular abnormalities, mainly manifesting as right ventricular dilation and/or dysfunction, caused by progressive fibrofatty replacement of the myocardium (16). ARVC very rarely manifests in childhood, and when it occurs is generally associated with worse outcome (17). Due to its rarity, several gaps in knowledge still exist. In their clinically focused review, Te Riele et al. describes the spectrum of childhood ARVC, the genetic architecture of the disease, and current diagnostic and therapeutic strategies.

## Restrictive cardiomyopathy

RCM is a myocardial disease characterized by abnormal diastolic function with restrictive physiology and normal ventricular diameter, wall thickness and systolic function (1). While two thirds of children have pure RCM phenotype, the remaining part has a mixed RCM-HCM phenotype (18). Children with RCM present the worst outcomes among any paediatric CMP group. In their review, Ditaranto et al. summarized the causes of childhood RCM, their pathophysiology, clinical presentation, and management.

## Left ventricular non-compaction cardiomyopathy

LVNC is a myocardial disease characterized by prominent left ventricular trabeculae and deep intertrabecular recesses (1). LVNC is a very heterogeneous condition ranging from asymptomatic cases to severely affected individuals with need for transplantation or at risk for sudden cardiac death. Schultze-Berndt et al. described the genotype-phenotype correlation and clinical outcome of a cohort of 149 patients (both paediatric and adult) with LVNC. They found that reduced left ventricular systolic function was the main independent predictor for adverse events.

Finally, next to articles purely focused on CMPs, Sarquella-Brugada et al. provide a comprehensive description of genotype-phenotype correlation of all rare variants in TRDN leading to malignant arrhythmias in paediatric patients.

The editors hope that readers of this Research Topic will find it of interest.
